# A Bibliometric and Citation Network Analysis of Myopia Genetics

**DOI:** 10.3390/genes12030447

**Published:** 2021-03-21

**Authors:** Cristina Alvarez-Peregrina, Clara Martinez-Perez, Cesar Villa-Collar, Miguel Ángel Sánchez-Tena

**Affiliations:** Faculty of Biomedical and Health Sciences, Universidad Europea de Madrid, 28670 Madrid, Spain; cristina.alvarez@universidadeuropea.es (C.A.-P.); villacollarc@gmail.com (C.V.-C.); masancheztena@gmail.com (M.Á.S.-T.)

**Keywords:** genetic, myopia, citation network

## Abstract

Background: To aim of the study was describe the growth of publications on genetic myopia and understand the current research landscape through the analysis of citation networks, as well as determining the different research areas and the most cited publications. Methods: The Web of Science database was used to perform the publication search, looking for the terms “genetic*” AND “myopia” within the period between 2009 and October 2020. The CitNetExplorer and CiteSpace software were then used to conduct the publication analysis. To obtain the graphics, the VOSviewer software was used. Results: A total of 721 publications were found with 2999 citations generated within the network. The year 2019 was singled out as a “key year”, taking into account the number of publications that emerged in that year and given that in 2019, 200 loci associated with refractive errors and myopia were found, which is considered to be great progress. The most widely cited publication was “Genome-wide meta-analyses of multiancestry cohorts identify multiple new susceptibility loci for refractive error and myopia”, an article by Verhoeven et al., which was published in 2013. By using the clustering function, we were able to establish three groups that encompassed the different research areas within this field: heritability rate of myopia and its possible association with environmental factors, retinal syndromes associated with myopia and the genetic factors that control and influence axial growth of the eye. Conclusions: The citation network offers a comprehensive and objective analysis of the main papers that address genetic myopia.

## 1. Introduction

Myopia is the leading cause of visual impairment worldwide and there are both genetic and environmental factors that contribute to its development [1]. Efforts to decipher the hereditary determinants of myopia began in the 1960s, and these were carried out in studies of monozygotic twins. These studies demonstrated that myopia is hereditary, with a rate of 91%, and that genes explain up to 80% of the variances that exist in terms of refractive error [2,3,4,5]. Until the advent of genome-wide association studies (GWAS), studies using linkage analysis in families or investigating variants in candidate genes were conducted to identify disease-associated genes. However, they were not successful in myopia, and until 2009, there were no genes known for common myopia that occur in the general population. GWAS allowed the identification of many refractive error genes associated with myopia. Thus, knowledge about the molecular machinery underlying myopia was increased as well as promising clues for the development of future therapies [6].

During the last decade, different consortia such as the International Consortium for Refractive Error and Myopia (CREAM), the 23andMe Research Team and the UK Biobank Eye and Vision Consortium have tried to identify the genetic variants that are associated with different refractive errors, mainly myopia. In 2013, 39 SNP (single nucleotide polymorphism) mutations were found to be associated with myopia [7,8]. In 2016, Tideman et al. [9], as members of the CREAM, analyzed the influence of these SNPs on axial length and corneal radius (AL/CR) as a function of age. In 2018, a meta-analysis involving 160,420 subjects increased the number of genetic polymorphisms that were associated with refractive errors from 39 to 161 [10]. In the most recent large-scale study that was published in *Nature Genetics* in 2020, Hysi et al. [11] conducted a meta-analysis of genome-wide association studies (GWAS). Their study involved a total of 542,934 European participants, and 336 new genetic loci associated with refractive error were identified. Citation network analysis is used when we want to look for a specific topic within the scientific literature. By analyzing one publication, we can discover other relevant publications, with the objective being to show, in a qualitative and quantitative manner, any connections that may exist between articles and authors through the creation of groups [12]. This method also allows for the quantification of the most commonly cited publications from each group, and, likewise, a specific research field can be developed or, where necessary, the literature search can concentrate on a particular topic [12,13].

In recent years, citation analysis, the exploration of reference patterns in both academic and scientific literature, has been applied in order to analyze the impact of research, knowledge flows and knowledge networks. It is also important in information science, mainly in the representation of knowledge and in the retrieval of information. Recently, there has been an increase in interest in citation analysis in order to solve questions related to research, management or information services (evaluation of research or visualization of knowledge).

This interest arises from the increase in the availability and accessibility of digital bibliographic data (both citations and full text) as well as in the relevant computer technologies. This provides a wealth of data and the tools necessary for researchers to reliably perform citation analyses on a large scale, even without having access to special data collections [12,13].

Therefore, taking into account the rising number of publications on genetic myopia, the objective of this study was to determine the different areas of research as well as the most commonly cited publication. Likewise, by using the CitNetExplorer software, a program used to assess the development of scientific research within a specific field, we looked to evaluate the existing relationships between the publications and the different research groups.

## 2. Materials and Methods

### 2.1. Database

We used the Web of Science (WoS) database to conduct our search, establishing the following search terms: “myopia” and “genetic.” We selected these terms as they are the two most frequently used terms in all of the fields of research, therefore meaning that they were in line with the objectives of this study.

Taking into consideration the fact that the search results had articles in common, we applied the Boolean operator AND and the wildcard operator “*” in order to look for both the singular and plural forms of the words. As a result, the search term that was employed was: “genetic*” AND “myopia.” Additionally, the search was also conducted by choosing the Subject as the search field, before going on to limit the results according to the abstract, keywords and title. This search covered the time frame from 2009 to October 2020.

With regard to the citation indexes, the Social Sciences Citation Index, the Science Citation Index Expanded and the Emerging Sources Citation Index were used. The publications were searched and downloaded on 20 October 2020.

The study was favorably evaluated by the ethics investigation committee of Universidad Europea de Madrid (CEI-UE) under the code CIPI/19/102. In addition, the study was developed in accordance with the standards recognized by the Declaration of Helsinki by the World Medical Association (64th General Meeting, Fortaleza, Brazil, October 2013). Informed consent was not necessary.

### 2.2. Data Analysis

The CitNetExplorer software was used for the publication analysis; this software enables the researcher to both analyze and visualize the citation networks of scientific publications. Citation networks can also be downloaded directly from the Web of Science, and likewise, it is possible to manage citation networks that are comprised of millions of publications and other related citations.

The citation score attribute was used to conduct a quantitative analysis of the most mentioned publications within a specific period (self-citations were excluded). By doing so, it was possible to quantify not only the internal connections within the Web of Science database but also any external connections as well, which meant, therefore, that other databases were also taken into consideration [13].

The CitNetExplorer offers a number of techniques that can be used when analyzing publications’ citation networks. The clustering functionality is achieved by using the formula that was established by Van Eck in 2012 [13].

(1) $$V\left(c_{1\;},\ldots ,c_{n}\right)=\underset{i<j}{\sum }\delta \;\left(c_{i},c_{j}\right)\left(s_{ij}-\gamma \right)$$

The clustering function was used to assign a group to each publication. By using this function and taking into consideration the results from the citation networks, it was possible to group publications with a greater level of association [13].

Finally, the Identifying Core Publications functionality was used to evaluate the core publications. This function serves to identify any publications that are considered to be at the core of a citation network, while disposing of any that are considered insignificant. When establishing the number of connections, we took into account the fact that the higher the value is of this parameter, the lower the number of core publications is [13]. This study took into consideration any publications with four or more citations in the citation network. On the other hand, we also used the drilling down functionality to attain a more in-depth analysis of each of the groups at different levels.

The VOSviewer software, which allows for the visualization and creation of bibliometric networks, was used for creating the graphs. 

Scientometric analysis was conducted using the CiteSpace software (5.6.R2). This is a Java language-based software which is formed by five basic theoretical aspects: Kuhn’s model of scientific revolutions, Price’s scientific frontier theory, the organization of ideas, the best information foraging theory of scientific communication and the theory of discrete and reorganized knowledge units [14,15]. In the scientometric analysis process, some parameter indicators exist for a specific evaluation. The H index is a mixed quantitative index which is used to evaluate the quantity and level of academic output of researchers and institutions. The H index indicates that h of the N articles published in the journal have been cited at least h times [16]. The degree indicates the number of connections that exist between authors (institutions, countries) in the co-occurrence knowledge graph. In the case in which the degree value is higher, this suggests a greater level of communication and cooperation between the authors (institutions, countries). Besides, intermediary centrality is an indicator that is used to determine the importance of nodes in the research cooperation network, and the half-life is a parameter that is used to represents the continuity of institutional research from a time perspective [14].

Therefore, with this analysis, we obtained the articles with the largest citation networks and the most importance in each research area.

## 3. Results

In 1970, the first articles on genetic myopia were published; therefore, the selected period of study was from 1970 to October 2020. Following the WoS search, 983 publications were found according to title, abstract and keywords, as well as 6100 citation networks. 

**Figure 1:**
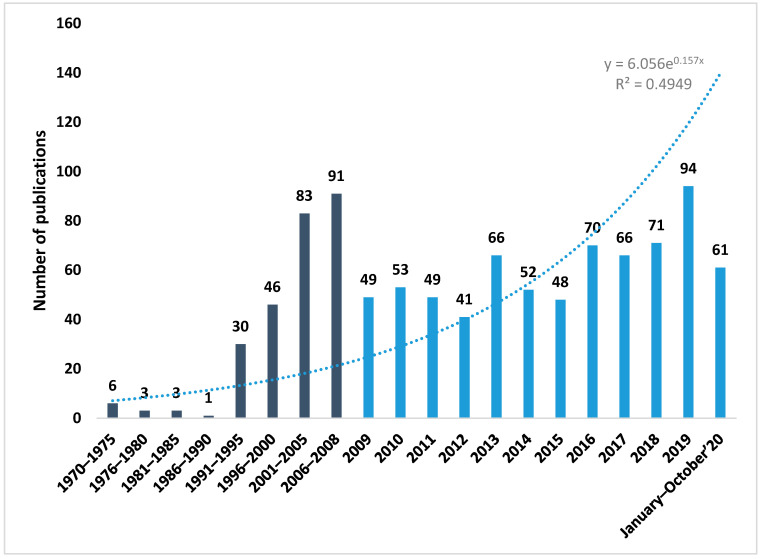
Number of publications per year.

As we can see in Figure 1, the number of publications on genetic myopia has increased exponentially since 2009 (1970–2008: 26.75%; 2009–October 2020: 73.27%). Notably, 2019 was the year with the highest number of publications: 94 publications and 20 citation networks. In turn, 2019 was also determined to be the “key year”, not only because of the number of publications but also the fact that 200 loci associated with refractive errors and myopia were found, which is a great advance.

For this reason, and according to the study conducted by de Tedja et al. [6], the selected time frame for performing the bibliometric and citation network analysis was from 2009 to October 2020. Before 2009, genes for common myopia were not known to occur in the general population.

### 3.1. Description of Publications

Of all the publications, 80.17% were articles, 11.37% were reviews, 6.10% were congress and conference abstracts, 1.39% were book chapters and the remaining 1% were “proceeding papers, letters, corrections and data papers”. According to van Wesel [17], the reasons why the number of articles is significantly higher may be due to alterations in the publication policy, the interest of the authors or the topics that are being covered.

#### 3.1.1. Language and Countries

With regard to the language of the publications, 98.21% were published in English, 1.37% in German, 0.27% in French and 0.14% in Korean. This is because English is one of the most widely used languages in the world; therefore, researchers who write in this language have a greater chance of having their work published [18].

Therefore, as shown in Supplementary Figure S1, the countries with the highest publication rate were the United States (31.18%), China (28.57%) and England (15.52%). Supplementary Figure S1 shows the most important publications and the group that they belong to. An item’s color represents the group to which it belongs and the lines between the elements represent the existing links.

Supplementary Table S1 shows the main characteristics of the six most important groups in Supplementary Figure S1.

**Figure 2:**
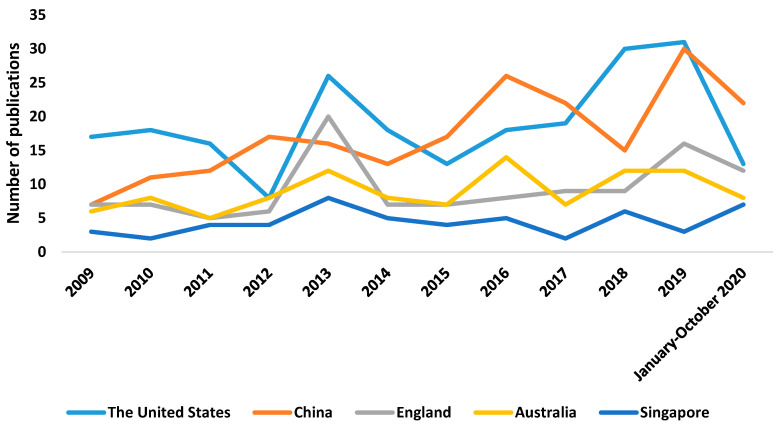
Number of publications per country and year.

Figure 2 and Table 1 show the trajectory of publications in the five countries that boast the highest number of articles on genetic myopia, and the highest number can be appreciated in the United States. The upward tendency in the number of publications from countries such as the United Kingdom or the United States may be due to a range of different factors, including the fact that these are anglophonic countries or the possible affiliations that exist between different research groups in the scientific community [19,20]. It should be considered that in our study, England stands out, not counting other countries of United Kingdom.

#### 3.1.2. Research Areas

Research on this topic is multidisciplinary. The fields of ophthalmology (60.30%) and hereditary genetics (19.23%) are particularly relevant (Table 2).

#### 3.1.3. Authors and Institutions

As shown in Table 3 and Supplementary Figure S2, the authors with the highest number of publications on genetic myopia were Guggenheim JA (6.59%), Hammond CJ (5.91%) and Saw SM (5.36%).

Supplementary Table S2 shows the main characteristics of the seven most important groups in Supplementary Figure S2.

**Figure 3:**
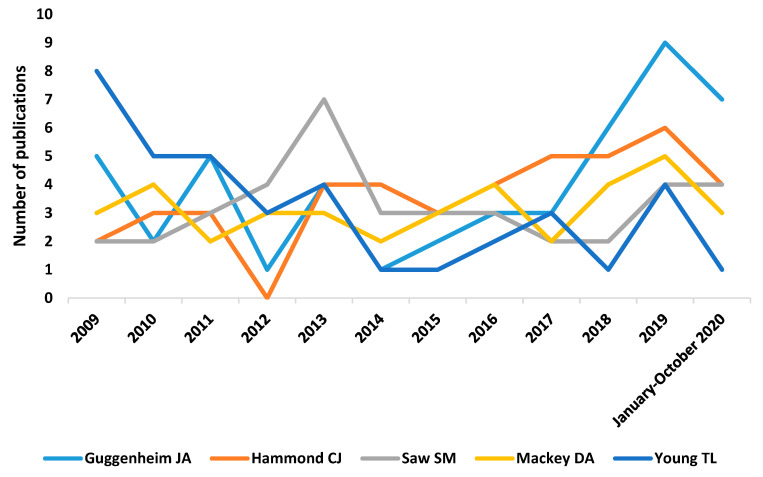
Number of publications per authors and year.

Figure 3 shows the trajectory of the five authors with the largest number of articles on genetic myopia, with Guggenheim JA’s trajectory considered to be the most relevant. 

The institutions with the highest number of publications, as indicated in Table 4 and Supplementary Figure S3, were Sun Yat-sen University (8.10%), University of Melbourne (7.69%) and Kings College London (7.01%).

Supplementary Table S3 shows the main characteristics of the six most important groups from Supplementary Figure S3. 

**Figure 4:**
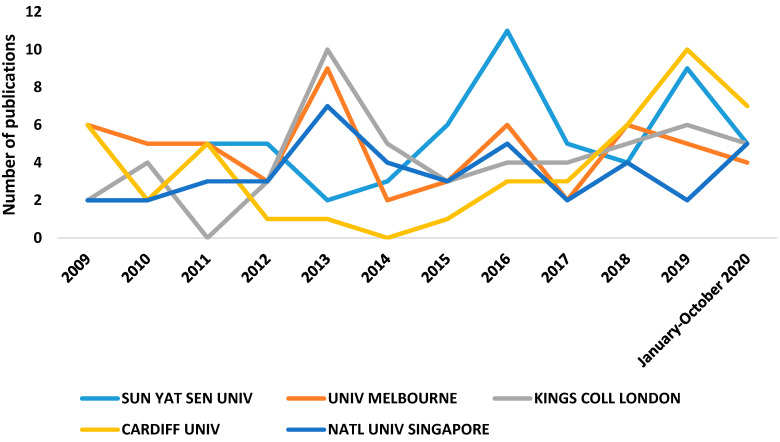
Number of publications per institutions and year.

Figure 4 shows the trajectory of the five institutions with the largest number of articles on genetic myopia, with the most relevant being Sun Yat-sen University. It also shows that there has been a significant increase in publications at Cardiff University in recent years.

#### 3.1.4. Journals

Table 5 and Supplementary Figure S4 show the main journals that have published content on genetic myopia, indicating the number of publications that have been found in the WoS database. Most of the journals with a high publication rate and a high impact factor were from the United States and the United Kingdom.

Supplementary Table S4 shows the main characteristics of the six most important groups in Supplementary Figure S4.

**Figure 5:**
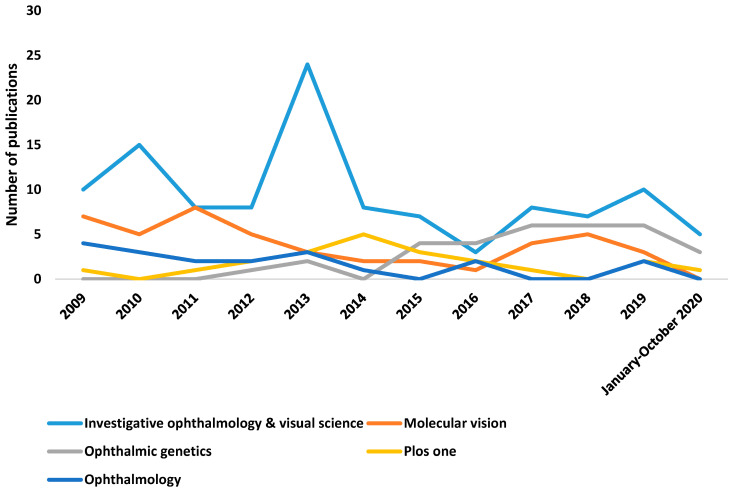
Number of publications per journals and year.

Figure 5 shows the trajectory of the five journals with the highest number of articles on genetic myopia, with a greater relevance of the journal *Investigative Ophthalmology & Visual Science.*

#### 3.1.5. Keywords

**Figure 6:**
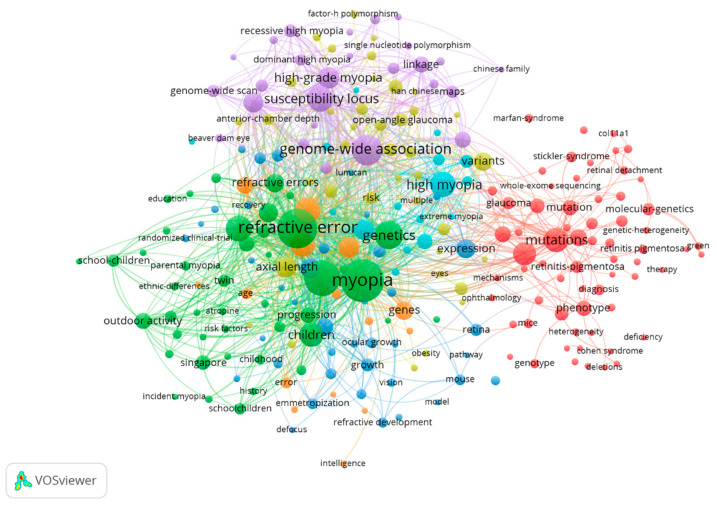
Connection between keywords.

The most used keywords were “Myopia” (215 publications), “Refractive error” (186 publications) and “Prevalence” (152 publications). Table 6 and Figure 6 show the most commonly used keywords in the most relevant publications.

Table 7 shows the main characteristics of the five most important groups in Figure 6. 

### 3.2. The Most Cited Publications 

Between 2009 and October 2020, 721 publications and 2999 citation networks were found. Table 8 shows the 20 most cited articles. 

The most cited publication was the article by Verhoeven et al. [21], which was published in 2013 and which had a citation index of 106. A genome-wide meta-analysis was performed, which included 37,382 individuals from 27 studies of European ancestry and 8376 from five Asian cohorts. Sixteen new loci for refractive errors in individuals of European ancestry were found (eight were shared with Asian). 

These new loci included candidate genes with functions in neurotransmission *(GRIA4*), ion transport (*KCNQ5*), retinoic acid metabolism *(RDH5*), extracellular matrix remodeling (*LAMA2* and *BMP2*), and eye development (*SIX6* and *PRSS56*). Previously reported associations with *GJD2* and *RASGRF1* were also found. Subjects with the highest genetic load shown a tenfold increased risk of myopia in a risk score analysis using associated SNPs. These results considerably advance the understanding of the mechanisms involved in refractive error and myopia. 

When analyzing the 20 most cited articles, all of them analyzed the heritability rate of myopia. Likewise, these articles also considered the contribution of genes and the environment in the development of refractive errors. 

### 3.3. Clustering

By using the clustering function, we were able to assign each publication in the citation network to a group, meaning, therefore, that the publications that are close to each other within the citation network tend to be in the same group. Therefore, each group consists of publications that are strongly linked to each other in terms of citation networks. In this way, it is possible to interpret that each group represents a particular topic within the scientific literature. In order to distinguish between the different groups, they were each assigned different colors, and the links between groups are shown by colored lines. 

**Figure 7:**
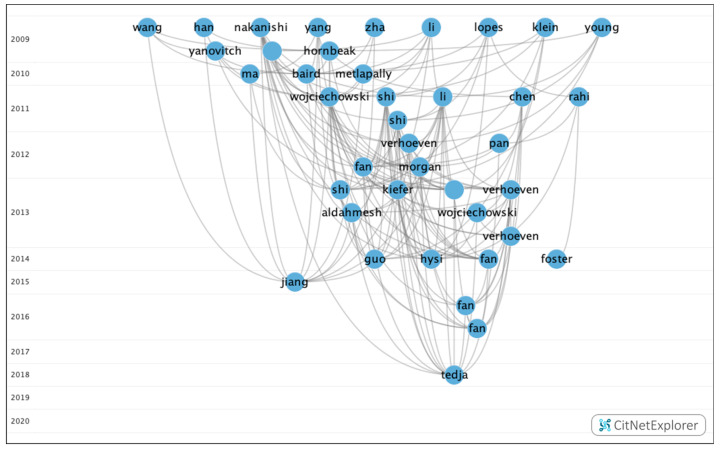
Citation network about genetic myopia.

Through this analysis, five groups were identified, of which three contained a significant number of publications (Figure 7). However, the remaining groups accounted for just 3.75%.

Table 9 shows the information on the citation networks for the three main groups, listed by size from the biggest to the smallest. 

**Figure 8:**
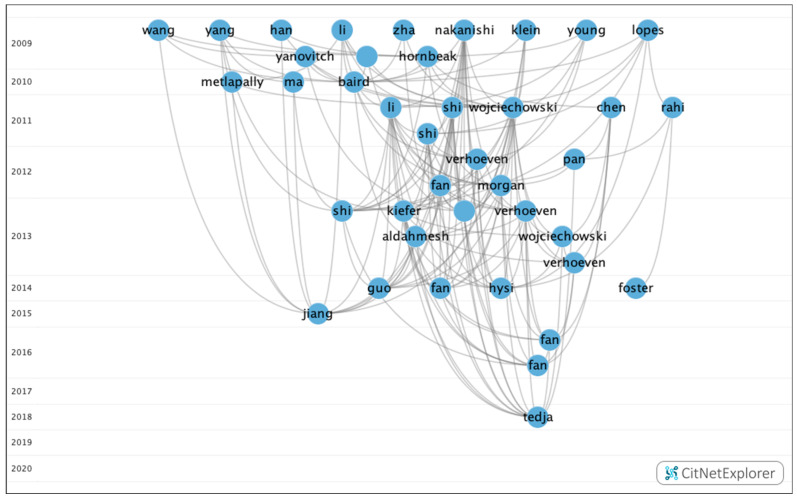
Citation network in group 1.

In group 1, there were 379 publications and 2579 citations within the network. The most commonly cited publication was the article by Verhoeven et al. [21], which was published in 2013 in *Nature Genetics* and which was also at the top of the list of the 20 most cited publications. The articles in this group evaluate the heritability of myopia and its possible association with the negative impact of environmental factors (Figure 8). 

In group 2, 54 publications and 95 citations were found within the network. The most commonly cited publication was the article by Sun et al. [39], which was published in 2015 in *Investigative Ophthalmology & Visual Science*. The aim of this study was to investigate mutations in 234 genes associated with retinal dystrophies in a cohort of 298 subjects with early-onset high myopia using whole-exome sequencing. The results showed that systematic analysis of variants in the 234 genes identified potential pathogenic mutations in 34 genes of 71 participants. Of these, 44 had mutations in 11 genes responsible for high myopia eye disease, including *COL2A1, COL11A1, PRPH2, FBN1, GNAT1, OPA1, PAX2, GUCY2D, TSPAN12, CACNA1F* and *RPGR*. The initial clinical records of the 71 patients with mutations did not show any diseases other than high myopia. In conclusion, mutations in genes responsible for retinal diseases were confirmed in a quarter of subjects with early onset of high myopia. 

**Figure 9:**
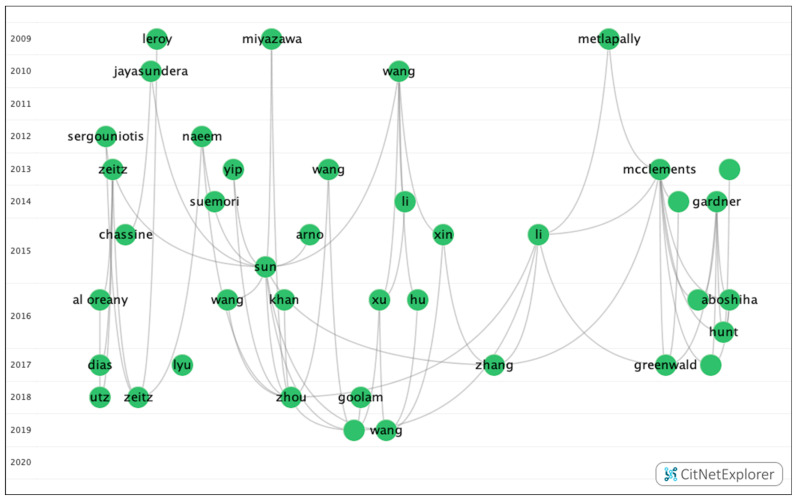
Citation network in group 2.

The articles in this group analyzed the allelic and genotypic frequencies that can lead to retinal syndromes associated with myopia (Figure 9). 

In group 3, 22 publications and 28 citations were found within the network. The most commonly cited publication was the article by Prashar et al. [40], which was published in 2009 in *Experimental Eye Research*. This study examines the relationship between eye size and body size in chickens from a genetic cross between a layer line (small body size and eye size) and a broiler line (large body and eye size). In total, 510 chickens were evaluated by keratometry and in vivo high-resolution A-scan ultrasonography at three weeks from birth. The diameter of the equatorial eye and the weight of the eye were measured after enucleation.

Changes in eye size parameters were explained by a multiple linear regression analysis of body weight (BW), body length (BL), head width (HW) and gender. 

Thus, the analysis of BW, BL, HW and sex predicted 51–56% of the variation in eye weight, axial length, corneal radius and equatorial eye diameter and 22% of the variation in lens thickness. When adjusting for sex, the three parameters of body size predicted between 45% and 49% of the variation in eye weight, axial length, corneal radius and eye diameter; however, they explained only 0.4% of the variation in lens thickness. 

**Figure 10:**
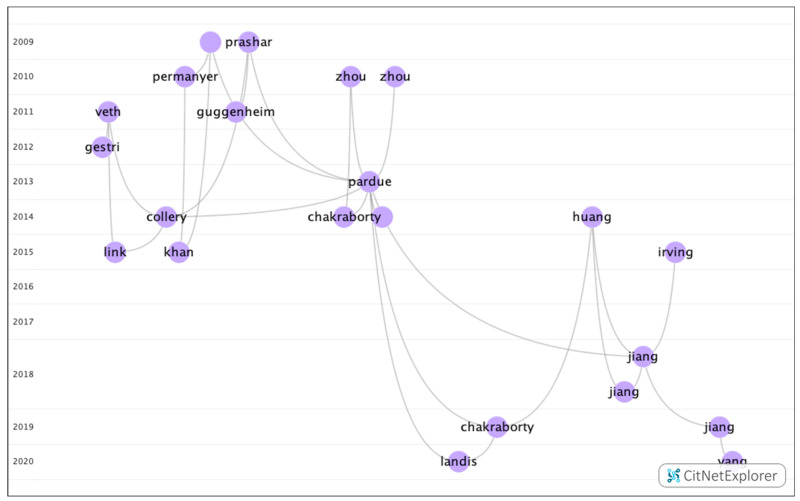
Citation network in group 3.

In conclusion, the variation in the eye size of chickens in this broiler–layer advanced intercross line is unlikely to be determined by pleiotropic genes that also influence body size. Therefore, in general, to understand the genetic determination of eye size, mapping the quantitative trait loci (QTL) that determine body size may be helpful (Figure 10). 

**Figure 11:**
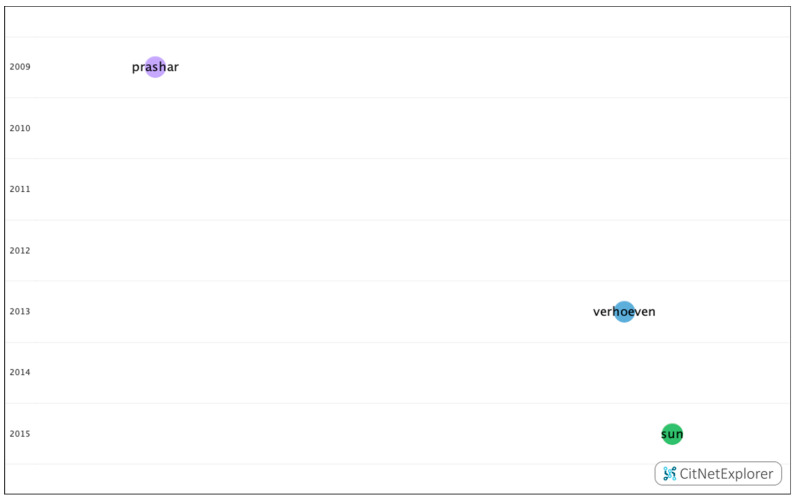
Relationship between the three main groups.

When analyzing the relationship between groups, it was determined that there was no connection between the groups. Therefore, each group analyzed clearly different topics (Figure 11). 

### 3.4. Core Publications

**Figure 12:**
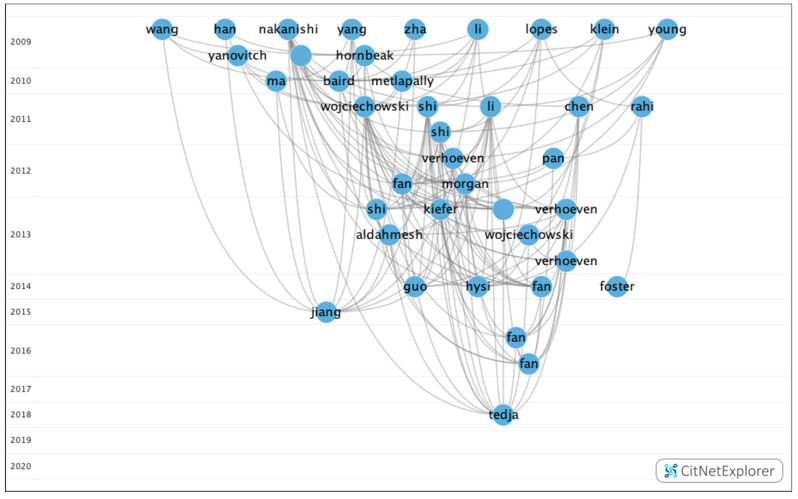
Core publications in citations network about genetic myopia.

In total, 353 publications with four or more citations were found and the citation network was 2696, representing 48.96%. This means that there is a clear focus on the research that is being carried out in this field. In this analysis, the main topic is the heritability rate of myopia, as well as the contribution of genes and the environment in the development of refractive errors (Figure 12). 

## 4. Discussion

This study aimed to analyze the literature available on genetic myopia and understand the current research outlook by grouping publications together according to similarities in the investigated research topics. In order to do so, the Web of Science database was used. This is one of the most comprehensive databases, as it covers data dating back to the year 1900. Nevertheless, it is worth considering that only international journals that have undergone a rigorous selection process are accepted in the Web of Science. This database allows for citation networks to be created; however, when conducting a systematic review of all of the literature that exists on a subject, its usefulness is somewhat limited given that it does not offer a general overview of the connections that exist between the citations of a group of publications. For this reason, CitNetExplorer and CiteSpace software were used, as these computer programs allow researchers to visualize, analyze and scrutinize the citation networks of scientific publications. These programs offer a much more detailed analysis when creating citation networks compared to other databases such as Web of Science or Scopus [13]. In addition, for the methodology, we based ours on other studies of citation networks carried out by our research team [41,42,43].

The first studies on genetic myopia were based on linkage analysis, and these studies were limited to the identification of genetic variants with a large effect on myopia [44]. It should be noted that three studies provided evidence of a myopia locus on chromosome 11. For the first time, a genome-wide linkage analysis of 221 dizygotic twins from the UK identified the MPY7 locus at 40 cm on chromosome 11p13 as a locus susceptible to myopia [45]. However, another study showed marginal evidence of this connection in an independent group of 485 dizygotic twin pairs from the United Kingdom [46]. 

Finally, a genome-wide exploration of 36 white families with a mean SE of −4.0D also provided strong evidence for the linkage of a myopia locus on chromosome 11 [47].

However, given the limited number of genes that have been identified, the advent of GWAS studies in the 2000s has significantly improved knowledge of the genetic architecture of diseases. 

At the beginning, GWAS for myopia were performed as a dichotomic result (case–control). However, since myopia constitutes a dichotomization of quantitative trait spherical equivalent, considering the quantitative trait should be more informative for gene mapping. Thus, in 2010, the first GWAS for spherical equivalent were conducted in 4000 participants [48,49]. The first loci to reach the genome-wide significance threshold were markers near the *RASGFR1* gene at 15q25.1 and near *GJD2* at 15q14. 

These early studies demonstrated the difficulty in mapping many genes using the spherical equivalent, which is what led to the creation of the Consortium for Refractive Error and Myopia (CREAM) in 2010, which brought together researchers and cohorts from the US, Europe, Asia and Australia. This is consistent with the results obtained in our study and it shows the growing interest in the importance of genetics in myopia on a global level. Thus, in the last decade, there has been an increase in publications from European and Asian countries with a rate of 50.72% and 17.83%, respectively. Therefore, although academic exchanges and cooperation do exist between authors, institutions and countries in the field of myopia, these collaborations tend to occur between different institutions within a particular country or between influential academics within an institution. In this field, although developed countries such as England, the US and Australia, etc., remain in the lead, more and more developing countries are beginning to present a relatively high number of articles and distinguished research institutions. Thus, Sun Yat-Sen University in China stands out in this area of research, with a publication rate of 8%. In turn, in 2015, the International Myopia Institute was created; this institute was formed by experts from all over the world who have come together with the aim of advancing research, patient management and education in myopia. The aim of this initiative is to prevent future vision problems and blindness associated with increased cases of myopia by organizing meetings between scientists, doctors, legislators, governments and educators in the field of myopia in order to stimulate collaboration and the exchange of knowledge [50]. At the same time, the study by Tedja et al. [6], which was published in the year 2019, which has been identified as a “key year”, is considered to be particularly relevant, and said study conducted an exhaustive search of the existing literature on common refractive error, high myopia and myopia-associated syndromes. The result showed nearly 200 genetic loci associated with refractive error and myopia. Furthermore, it was determined that the risk variants mostly carry low risk but are highly prevalent in the general population. In turn, they concluded that several genes for syndromic secondary myopia overlap with those for common myopia. The polygenic risk scores showed overrepresentation of high myopia in the higher deciles of risk. The annotated genes have a wide variety of functions and all retinal areas appear to be sites of expression. Another relevant study was the one conducted by Pozarickij et al. [51], in which they analyzed gene–environment or gene–gene interactions in myopia. To do this, they tested the hypothesis that the variants associated with refractive error exhibit heterogeneity in effect size, a distinctive characteristic of genetic interactions. Of the 146 variants tested, evidence of non-linear and non-uniform effects was found in 66 at Bonferroni-corrected significance (*p* < 1.1×10^−4^) and 128 at nominal significance (*p* < 0.05). The *LAMA2* rs12193446 variant had an effect size varying in different individuals from −0.20 diopters (95% CI: −0.18 to −0.23) to −0.89 diopters (95% CI: 0.71 to −1.07). At the extremes of the phenotype, SNP effects were strongest and weakest in emmetropes. One explanation for this finding was that gene–environment or gene–gene interactions in myopia are omnipresent. In turn, with the rise of large biobanks such as the UK Biobank [5,52], further GWAS meta-analyses between large consortia and companies will allow for the identification of many more genes. This will allow the molecular mechanism of myopia genesis to be fully obtained. 

For this reason, given the increase in the prevalence of myopia on a global level and the fact that this varies geographically, a greater exchange of scientific research between countries will make it possible for knowledge to be built on the genes associated with myopia based on the different environmental factors [53]. 

In terms of the journal with a high number of publications on genetic myopia, *Investigative Ophthalmology & Visual Science* is worth mentioning, as this journal occupies the tenth position in the ophthalmology category with an impact factor of 3.47. Articles were published in 26 journals in the ophthalmology and optometry topic category, which was to be expected given that genetic myopia is a specialty within the field of optometry and ophthalmology. The journal with the highest impact factor was *Ophthalmology*, 3.57. However, it is important to consider that although the impact factor is a critical index of a journal’s importance, it is not an absolute measure index, nonetheless. The main difference between the two indexes is that the latter is based not only on the impact of the research results but also on the authors’ physical and intellectual contributions [54].

Research on the genetics of myopia, genetic epidemiology and epigenetics is thriving, and this research is providing a wealth of new insights into the molecules that are involved in refractive myopia genesis [5]. To date, 904 genetic loci have been found associated with refractive error. In turn, it has been found that refractive error is genetically heterogeneous and that it is driven by genes that participate in the development of all the anatomical components of the eye. Genetic factors that control circadian rhythm and pigmentation have also been found to be involved in the development of myopia and refractive error [11]. 

GWAS studies have been very effective in evaluating the role of common variants in myopia; however, said methods cannot effectively characterize very rare genomic variants. Whole-exome sequencing (WES) allows for the investigation of rare variants in exon regions; however, due to the cost, applications to date have primarily been in studies of familial or early-onset high myopia studies [55,56,57,58]. The study by Kloss et al. [59], in which WES was performed on 14 high myopia families, identifying 104 genetic variants in both known MYP loci as well as in new loci is worth mentioning. In familial studies, most variants showed an autosomal dominant mode of inheritance [26,37,59,60], although heterozygous X-linked mutations were found in ARR3 [61]. 

However, despite progress, the chain of events that form the myopia signaling cascade and the triggering for scleral remodeling are still largely unknown. The next steps should include all of the technological advances necessary to analyze complex disorders, such as the expansion of omics (genomics, transcriptomics, proteomics and metabolomics) and the use of multi-source study populations, environmental genomics and systems biology in order to organically integrate the findings and improve our understanding of the development of myopia in a quantitative way through big data analysis, in combination with the expansion of omics and other approaches (deep learning or artificial intelligence) [6]. 

Therefore, in the coming years, it is anticipated that there will be a significant increase in the number of genes related to myopia, and this will allow for the prediction of refractive error and the development of personalized strategies for the prevention of myopia in the future. 

## 5. Conclusions

In conclusion, this study offers a comprehensive and objective analysis of the main existing studies on genetic myopia. In addition, by using the Web of Science database and the CitNetExplorer software, it was possible to view, analyze and explore the most cited articles and the existing citation networks to date.

Currently, 336 new loci associated with myopia have been discovered. The increase in biobanks will allow for more meta-analyses of GWAS among large consortia and companies, and therefore, many more genes will be identified. In this way, the molecular mechanism of myopia genesis will be fully understood.

Therefore, genetic myopia studies will provide new insights into the evolution of myopia and promising concepts for future therapies. However, the genetic architecture and its molecular mechanisms have yet to be clarified. The knowledge of genetic risk prediction models is improving; however, it must be expanded to have an impact on clinical practice. That is, the knowledge of the genetic influence will allow to establish the bases on the inheritance and the appearance of myopia as well as improve the effectiveness of the treatment methods that currently exist.

## Figures and Tables

**Table 1:** The five countries with the highest number of publications.

Country	Publications (%)	Centrality	Degree	Half-Life
The United States	225 (31.18%)	0.40	38	5.5
China	208 (28.57%)	0.02	19	6.5
England	113 (15.52%)	0.12	33	5.5
Australia	107 (14.70%)	0.19	33	5.5
Singapore	53 (7.28%)	0.05	25	5.5

**Table 2:** The 10 research fields with the highest number of publications.

Category	Frequency	Centrality	Degree	Half-Life
Ophthalmology	437	0.16	16	5.5
Hereditary genetics	138	0.05	15	7.5
Biochemistry molecular biology	78	0.10	19	4.5
Science technology other topics	53	0.08	3	7.5
Experimental medicine research	31	0.00	29	5.5
General internal medicine	25	0.00	3	6.5
Neurosciences neurology	18	0.07	15	5.5
Pediatrics	8	0.03	11	7.5
Pharmacology and pharmacy	7	0.02	4	7.5
Psychology	7	0.03	10	3.5

**Table 3:** The 10 authors with the largest number of publications.

Author	Number of Publications	*H* Index	Total Citations	Citation Average	Centrality	Degree
Guggenheim JA	48	33	4173	24.99	0.08	53
Hammond CJ	43	16	991	23.05	0.02	29
Saw SM	39	18	2047	52.49	0.04	48
Mackey DA	38	15	792	20.84	0.03	33
Young TL	38	22	1343	35.34	0.16	54
Hysi PG	31	12	649	20.94	0.02	37
Klaver CCW	30	13	590	19.67	0.02	38
Williams C	30	14	623	20.77	0.02	41
Wojciechowski R	30	16	923	30.77	0.00	15
Zhang QJ	29	15	474	16.34	0.04	15

**Table 4:** The 10 institutions with the largest number of publications.

Category	Frequency	Centrality	Degree	Half Life
Sun Yat-sen University	59	0.03	20	6.5
University of Melbourne	56	0.04	40	4.5
Kings College London	51	0.05	52	5.5
Cardiff University	45	0.03	49	8.5
National University of Singapore	42	0.04	58	5.5
University of Western Australia	37	0.06	41	5.5
Erasmus MC	36	0.03	64	5.5
National Human Genome Research Institute	34	0.02	30	5.5
University College de Londres	34	0.08	42	5.5
University of Pennsylvania	33	0.01	31	3.5

**Table 5:** The 10 journals with the largest number of publications.

Journal	Total Publications	Impact Factor (2019)	Quartile	SJR (SCImago Journal Rank) (2019)	Citations /Docs (2 years)	Total Citations (2019)	Centrality	*H* Index	Country
Investigative Ophthalmology & Visual Science	113	3.47	Q1	1.79	3.458	8592	0..00	209	United States
Molecular Vision	45	2.20	Q2	0.86	2.213	724	0.00	88	United States
Ophthalmic Genetics	32	1.31	Q4	0.63	1.336	411	0.00	38	United Kingdom
PLOS One	21	2.74	Q2	1.02	2.942	193,380	0.00	300	United States
Ophthalmology	19	8.47	Q1	4.41	8.476	6778	0.00	229	Netherlands
Scientific Reports	19	3.99	Q1	1.34	4.149	283,384	0.00	179	United Kingdom
Experimental Eye Research	17	3.01	Q1	1.14	3.233	2169	0.00	119	United States
Acta Ophthalmologica	14	3.36	Q1	1.42	3.304	2369	0.00	82	United States
British Journal of Ophthalmology	14	3.61	Q1	1.89	4.026	3591	0.00	146	United Kingdom
Optometry and Vision Science	14	1.46	Q3	0.89	1.789	1011	0.00	92	United States

**Table 6:** The 30 most used keywords.

Keyword	Frequency	Centrality	Degree	Total Link Strength
Myopia	215	0.03	40	1394
Refractive error	186	0.05	65	1397
Prevalence	152	0.05	57	1053
Genome-wide association	110	0.03	51	807
Genetics	94	0.07	56	609
Susceptibility locus	77	0.05	55	608
Risk factors	77	0.04	54	581
High myopia	73	0.06	56	485
Population	73	0.05	55	484
Heritability	70	0.02	36	488
Mutations	68	0.04	35	329
Children	67	0.03	40	432
Gene	60	0.06	47	302
High-grade myopia	54	0.07	64	446
Eye	50	0.05	45	311
Axial length	49	0.04	49	379
Association	48	0.09	60	321
Form-deprivation myopia	46	0.03	42	319
Ocular refraction	45	0.03	47	377
Visual impairment	44	0.06	55	358
Expression	43	0.09	50	255
Variant	39	0.05	38	38
Environment	33	0.07	59	246
Eye growth	33	0.04	48	227
Identification	33	0.04	35	162
Outdoor activity	32	0.02	41	250
Locus	32	0.02	31	207
Linkage	31	0.02	39	243
Family	30	0.08	49	145
Epidemiology	29	0.06	46	211

**Table 7:** Characteristics of the most used keywords.

Cluster	Color	Main Keywords	Topic	%
1	Red	mutations, gene, family, phenotype, identification	Genetic mutations	27.53
2	Green	prevalence, myopia, refractive error, genetics, risk factors	Prevalence of myopia in children and its risk factors	17.96
3	Blue	growth, eye growth, form-deprivation myopia, retina, expression	Axial length growth	15.92
4	Yellow	variants, macular degeneration, metanalysis, single nucleotide polymorphism, common variants, open-angle glaucoma	SNPs (Single Nucleotide Polymorphism) and genes related to myopia	15.92
5	Violet	genome-wide association, susceptibility locus, high-grade myopia, linkage, locus	Genome association	10.61

**Table 8:** The 20 most mentioned articles.

Author	Title	Journal	Year	Citation index
Verhoeven et al. [21]	Genome-wide meta-analyses of multiancestry cohorts identify multiple new susceptibility loci for refractive error and myopia	*Nat Genet*. 2013 Mar;45(3):314–8.	2013	106
Wojciechowski et al. [22]	Nature and nurture: the complex genetics of myopia and refractive error	*Clin Genet*. 2011 Apr;79(4):301–20	2011	85
Kiefer et al. [8]	Genome-Wide Analysis Points to Roles for Extracellular Matrix Remodeling, the Visual Cycle, and Neuronal Development in Myopia	*PLoS Genet*. 2013;9(2):e1003299.	2013	83
Morgan et al. [23]	Myopia	*Lancet*. 2012 May 5;379(9827):1739–48	2012	80
Nakanishi et al. [24]	A genome-wide association analysis identified a novel susceptible locus for pathological myopia at 11q24.1	*PLoS Genet*. 2009 Sep;5(9):e1000660	2009	59
Lopes et al. [25]	Estimating Heritability and Shared Environmental Effects for Refractive Error in Twin and Family Studies	*Invest Ophthalmol Vis Sci.* 2009 Jan;50(1):126–31	2009	55
Shi et al. [26]	Exome Sequencing Identifies ZNF644 Mutations in High Myopia	*PLoS Genet.* 2011 Jun;7(6):e1002084.	2011	54
Li et al. [27]	Genome-Wide Association Studies Reveal Genetic Variants in CTNND2 for High Myopia in Singapore Chinese	*Ophthalmology.* 2011 Feb;118(2):368–75	2011	47
Pan et al. [28]	Worldwide prevalence and risk factors for myopia	*Ophthalmic Physiol Opt.* 2012 Jan;32(1):3–16	2012	47
Shi et al. [29]	Genetic Variants at 13q12.12 are associated with high myopia in the Han Chinese population	*Am J Hum Genet.* 2011 Jun 10;88(6):805–813	2011	40
Hornbeak et al. [30]	Myopia genetics: a review of current research and emerging trends	*Curr Opin Ophthalmol*. 2009 Sep;20(5):356–62.	2009	38
Klein et al. [31]	Heritability Analysis of Spherical Equivalent, Axial Length, Corneal Curvature, and Anterior Chamber Depth in the Beaver Dam Eye Study	*Arch Ophthalmol.* 2009 May;127(5):649–55.	2009	38
Yang et al. [32]	Clinical and linkage study on a consanguineous Chinese family with autosomal recessive high myopia	*Mol Vis.* 2009;15:312–8	2009	36
Fan et al. [33]	Genetic Variants on Chromosome 1q41 Influence Ocular Axial Length and High Myopia	*PLoS Genet.* 2012;8(6):e1002753.	2012	35
Li et al. [34]	An International Collaborative Family Based Whole-Genome Linkage Scan for High-Grade Myopia	*Invest Ophthalmol Vis Sci.* 2009 Jul;50(7):3116–27	2009	34
Tedja et al. [10]	Genome-wide association meta-analysis highlights light-induced signaling as a driver for refractive error	*Nat Genet.* 2018 Jun;50(6):834–848.	2018	34
Aldahmesh et al. [35]	Mutations in LRPAP1 Are Associated with Severe Myopia in Humans	*Am J Hum Genet*. 2013 Aug 8;93(2):313–20	2013	33
Young [36]	Molecular genetics of human myopia: an update	*Optom Vis Sci.* 2009 Jan;86(1):E8-E22	2009	31
Jiang et al. [37]	Detection of Mutations in LRPAP1, CTSH, LEPREL1, ZNF644, SLC39A5, and SCO2 in 298 Families with Early Onset High Myopia by Exome Sequencing	*Invest Ophthalmol Vis Sci.* 2014 Dec 18;56(1):339–45	2014	31
Verhoeven et al. [38]	Large scale international replication and meta-analysis study confirms association of the 15q14 locus with myopia. The CREAM consortium	*Hum Genet.* 2012 Sep;131(9):1467–80	2012	30

**Table 9:** Information about the citation networks of the three main groups.

Main Cluster	Number of Publications	Number of Citation Links	Number of Citations Median (Range)	Number of Publications with ≥4 Citations	Number of Publications in the 100 Most Cited Publications
Group 1	379	2579	2 (0–107)	308	93
Group 2	54	95	1 (0–18)	0	5
Group 3	22	28	1 (0–10)	0	1

## Data Availability

Not applicable.

## Supplementary Materials

The following are available online at https://www.mdpi.com/2073-4425/12/3/447/s1, Figure S1: Collaboration between countries; Figure S2: Collaboration between authors; Figure S3: Collaboration between institutions; Figure S4: Collaboration between journals; Table S1: Characteristics of the main countries; Table S2: Characteristics of the main authors; Table S3: Characteristics of the main institutions; Table S4: Characteristics of the main journals.
