# Supplementary material for: A Bibliometric and Citation Network Analysis of Myopia Genetics
Source: Genes (Basel). 2021 Mar 21;12(3):447. doi: 10.3390/genes12030447 (PMC8003911; doi:10.3390/genes12030447)
Supplement: Supplementary file 1 [file genes-12-00447-s001.pdf]

Supplementary Material.

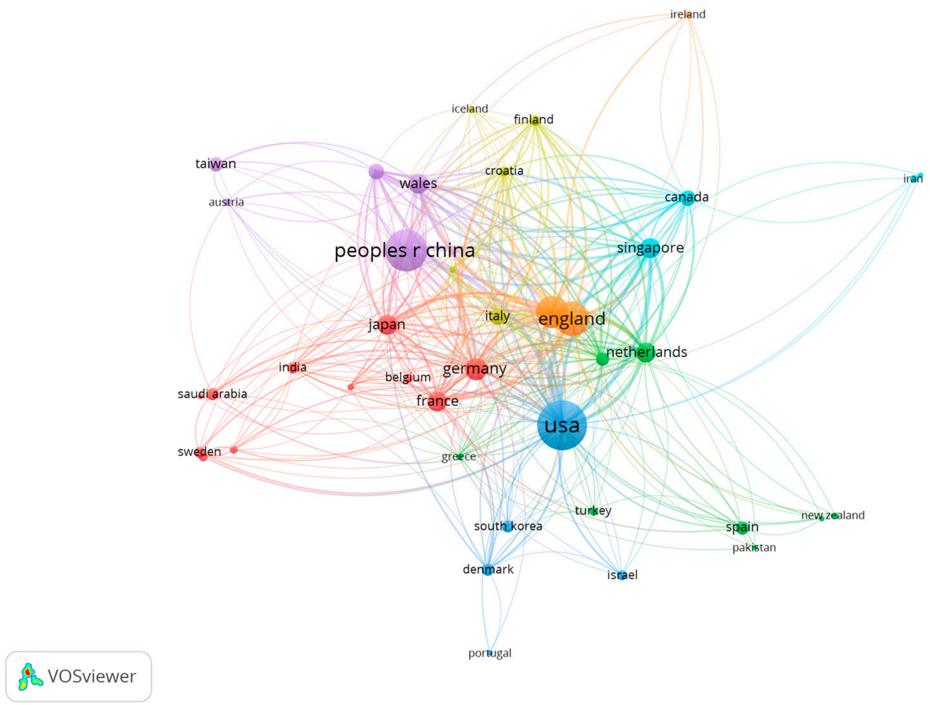

Supplementary figure S1. Collaboration between countries.

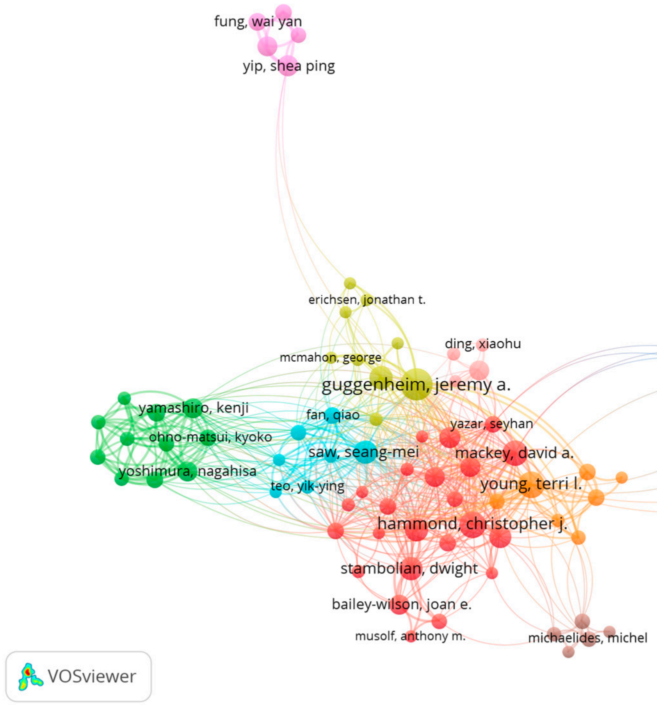

Supplementary figure S2. Collaboration between authors.

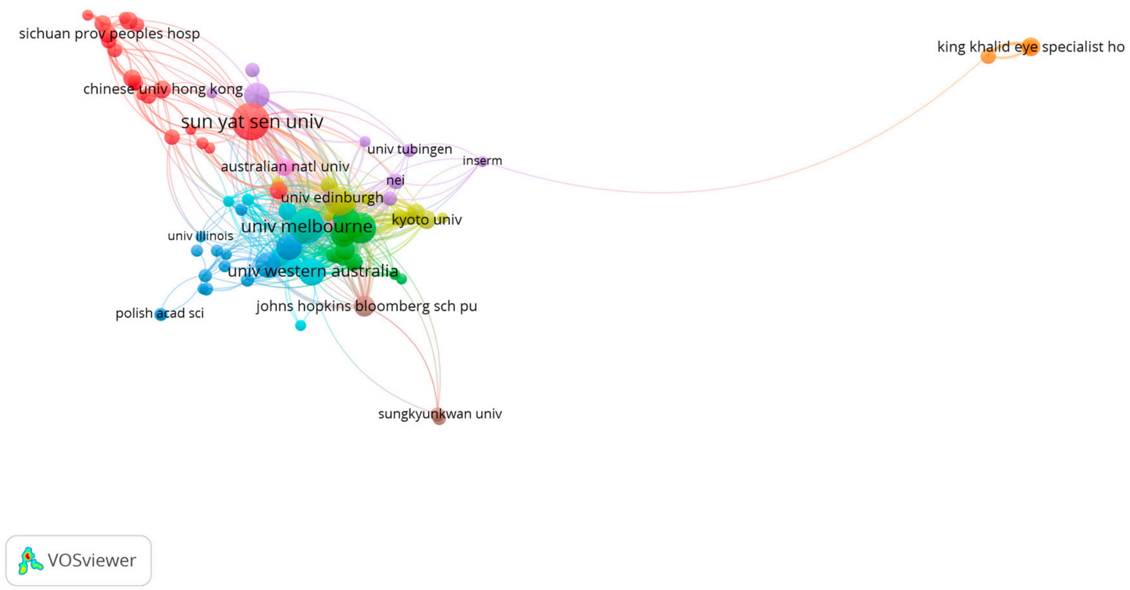

Supplementary figure S3. Collaboration between institutions.

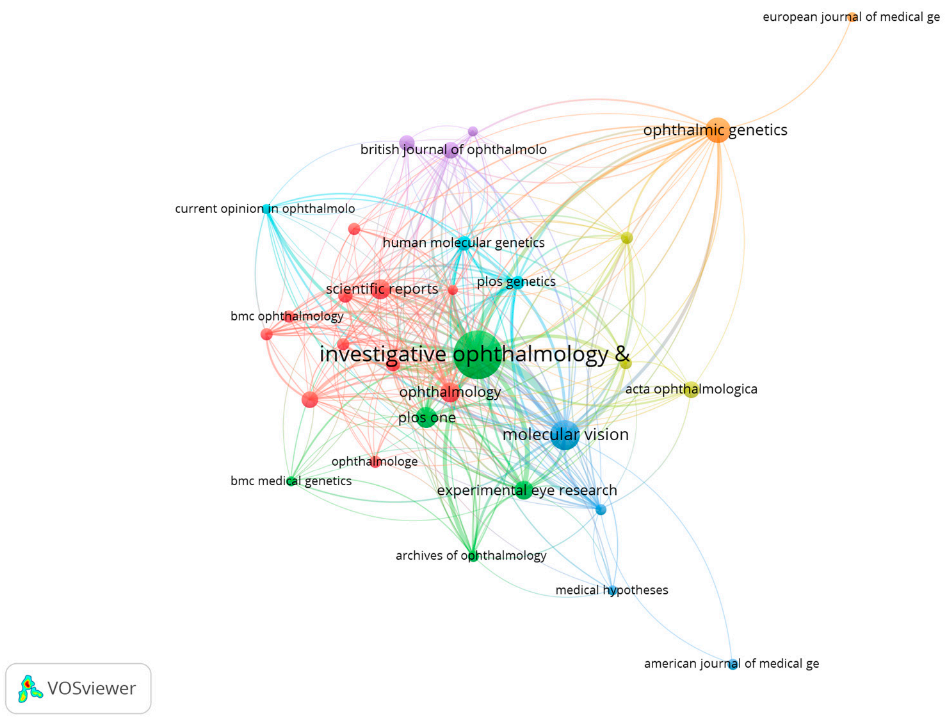

Supplementary figure S4. Collaboration between journals.

Supplementary table S1. Characteristics of the main countries.

Group	Color	Main Countries	Publications	Links
1st	Red	India	16	35
2nd	Green	Norway<br>Switzerland	53<br>20	245<br>122
3rd	Dark blue	The United States	225	396
4th	Violet	China	208	22
5th	Light blue	Singapore	53	237
6th	Orange	England	113	336

Supplementary table S2. Characteristics of the main authors.

Group	Color	Main authors	Publications	Links
1st	Red	Hammond C	43	89
		Wojciechowski R	30	121
2nd	Green	Mackey DA	38	110
3rd	Dark blue	Shi Y	12	32
4th	Yellow	Guggenheim JA	48	121
5th	Violet	Qinqjiong Z	28	102
6th	Light blue	Saw SM	39	82
7th	Orange	Young TL	38	111

Supplementary table S3. Characteristics of the main institutions.

Group	Color	Main institutions	Publications	Links
1st	Red	Sun Yat-sen University	59	69
2nd	Green	Kings College London	51	140
		Cardiff University	45	116
3rd	Dark blue	National Human Genome Research Institute	34	68
4th	Yellow	National University of Singapore	43	161
5th	Violet	The Hong Kong Polytechnic University	25	50
6th	Light blue	Melbourne University	56	150

Supplementary table S4. Characteristics of the main journals.

Group	Color	Main Journals	Publications	Links
1st	Red	Scientific reports	19	90
2nd	Green	Investigative Ophthalmology & Visual Science	113	606
3rd	Dark blue	Molecular vision	45	353
4th	Yellow	Acta Ophthalmologica	14	27
5th	Violet	British Journal of Ophthalmology	214	69
6th	Light blue	Plos one	21	193
